# Chemical Profiling and Cholinesterase Inhibitory Activity of Five *Phaedranassa* Herb. (Amaryllidaceae) Species from Ecuador

**DOI:** 10.3390/molecules25092092

**Published:** 2020-04-30

**Authors:** Raúl Moreno, Luciana R. Tallini, Cristina Salazar, Edison H. Osorio, Evelin Montero, Jaume Bastida, Nora H. Oleas, Karen Acosta León

**Affiliations:** 1 Group of Natural Products, Faculty of Pharmacy, University of Barcelona, Av. Joan XXIII, 27-31, 08028 Barcelona, Spain; raul.orlando.mm@gmail.com (R.M.); lucianatallini@gmail.com (L.R.T.); jaumebastida@ub.edu (J.B.); 2 Faculty of Pharmacy, Federal University of Rio Grande do Sul, Av. Ipiranga 2752, Porto Alegre RS 90610-000, Brazil; 3 Grupo de Investigación de Productos Naturales y Farmacia, Facultad de Ciencias, Escuela Superior Politécnica del Chimborazo, Panamericana Sur km 1 1/2, Riobamba EC060155, Ecuador; criss93danny@gmail.com (C.S.); eve_montero15@hotmail.com (E.M.); karen.acosta.leon@gmail.com (K.A.L.); 4 Facultad de Ciencias Naturales y Matemáticas, Universidad de Ibagué, Carrera 22 Calle 67, Ibagué 730001, Colombia; edison.osorio@gmail.com; 5 Centro de Investigación de la Biodiversidad y Cambio Climático (BioCamb) e Ingeniería en Biodiversidad y Recursos Genéticos, Facultad de Ciencias de Medio Ambiente, Universidad Tecnológica Indoamérica, Machala y Sabanilla, Quito EC170301, Ecuador

**Keywords:** alkaloids, Amaryllidaceae, cholinesterases, GC-MS, molecular docking, *Phaedranassa*

## Abstract

It is estimated that 50 million people in the world live with dementia, 60–70% of whom suffer from Alzheimer’s disease (AD). Different factors are involved in the development of AD, including a reduction in the cholinergic neurotransmission level. The Amaryllidaceae plant family contains an exclusive, large, and still understudied alkaloid group characterized by a singular skeleton arrangement and a broad spectrum of biological activities. The chemistry and biodiversity of Ecuadorian representatives of the *Phaedranassa* genus (Amaryllidaceae) have not been widely studied. In this work, five Ecuadorian *Phaedranassa* species were examined in vitro for their activity towards the enzymes acetyl- (AChE) and butyrylcholinesterase (BuChE), and the alkaloid profile of bulb extracts was analyzed by GC-MS. The species *Phaedranassa cuencana* and *Phaedranassa dubia* showed the most AChE and BuChE inhibitory activity, respectively. To obtain insight into the potential role of the identified alkaloids in these inhibitory effects, docking experiments were carried out, and cantabricine showed in silico inhibitory activity against both cholinesterase structures. Our results show that Amaryllidaceae species from Ecuador are a potential source of new drugs for the palliative treatment of AD.

## 1. Introduction

Natural products represent an interesting source of bioactive compounds and have led, directly or indirectly, to the development of about two thirds of the new medicines approved in recent years [1]. Plants are widely used in folk medicine, and plant extracts have long been tested in screening programs in pharmaceutical companies and university institutes as sources of new commercially viable drugs [2]. Alkaloids—secondary metabolites found mainly in plants—have an important role in drug development because of their structural diversity [1,3,4].

The alkaloids present in the Amaryllidaceae family have attracted considerable attention because of their unique and diversified structures, as well as their biological potential [5]. *Phaedranassa* Herb. is a small Amaryllidaceae genus of ten species, most of them endemic to Ecuador [6]. These species are found in specific localities restricted to dry valleys or wet slopes in the Andes over 1200 m [7]. Except for *P. dubia*, the seven endemic species from Ecuador are either endangered or vulnerable to extinction under IUCN criteria [8]. Studies have shown that the genetic structure of the species has been influenced by relatively recent events such as volcanism and urban development [9,10].

About 50 million people live with dementia in the world today, and Alzheimer’s disease (AD) is one of the most usual forms of this pathology [11]. Different factors seem to be involved in the development of AD, including a reduction in the cholinergic neurotransmission level [12,13]. At the beginning of this century, the Amaryllidaceae alkaloid galanthamine was approved by the Food and Drug Administration (FDA) for the clinical treatment of AD because of its acetylcholinesterase inhibitory activity [14]. As high cost and low yields render galanthamine synthesis inviable, it is obtained by pharmaceutical companies from natural sources such as *Galanthus nivalis*, *Leucojum aestivum*, *Lycoris radiata*, and several species of *Narcissus* [15].

**Figure 1:**
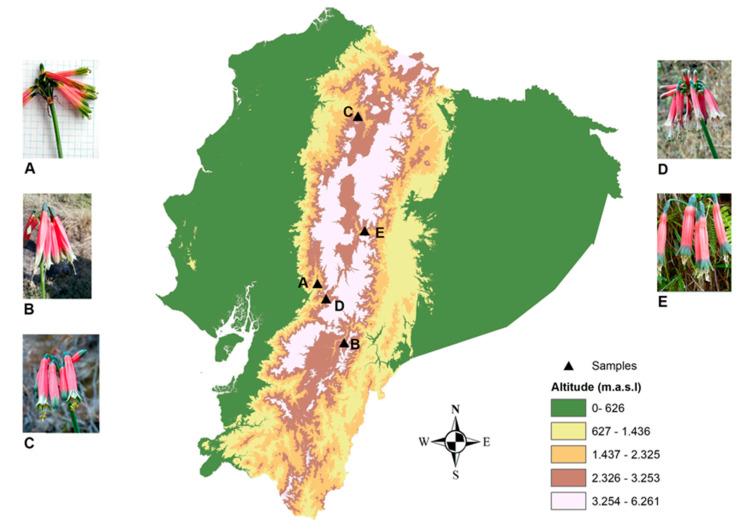
Distribution of *Phaedranassa* Herb. species in Ecuador. (**A**): *P. cinerea*; (**B**): *P. cuencana*; (**C**): *P. dubia*; (**D**): *P. glauciflora*; **E**: *P. tunguraguae*.

Considering the Amaryllidaceae alkaloid potential in AD therapy, the aim of this study was to identify the alkaloid content of five different *Phaedranassa* species collected in Ecuador (Figure 1) and to analyze the anticholinesterase activity of these samples. The alkaloid profiling of *P. cinerea*, *P. cuencana*, *P. dubia*, *P. glauciflora*, and *P. tunguraguae* was performed by gas chromatography coupled to mass spectrometry (GC-MS). The AD therapeutic potential of these species was evaluated by analyzing the AChE and BuChE inhibitory activity of each sample. Molecular docking studies were also carried out to investigate the binding affinity of the alkaloids identified in *Phaedranassa* species toward the active sites of AChE and BuChE.

## 2. Results and Discussion

### 2.1. Alkaloids Identified by GC-MS

**Figure 2:**
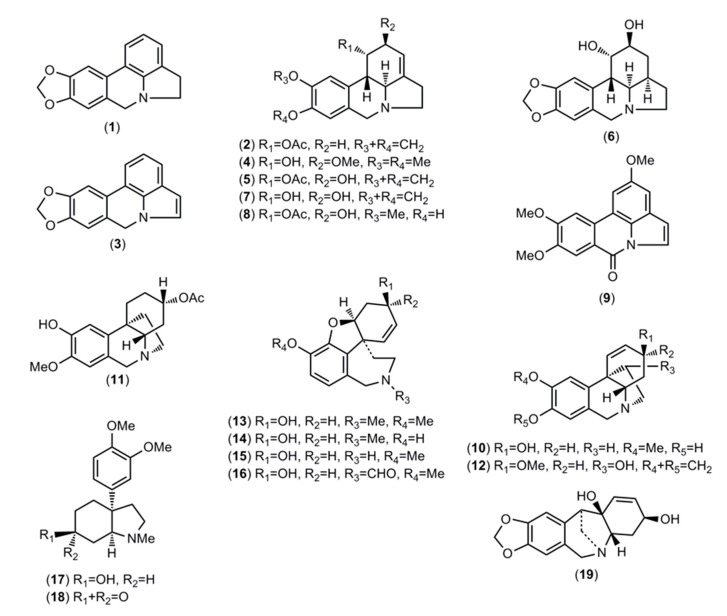
Alkaloids identified in Ecuadorian *Phaedranassa* Herb. by gas chromatography coupled to mass spectrometry (GC-MS).

The identified alkaloids and their structures are represented in Table 1 and Figure 2, respectively. The alkaloids present in the analyzed samples were identified by comparing their GC-MS spectra and Kovats retention index (RI) values with those of authentic Amaryllidaceae alkaloids previously isolated and identified by spectrometric methods (NMR, UV, CD, IR, MS) in the Natural Products Laboratory of Barcelona University, the NIST 05 Database, or literature data.

**Figure 3:**
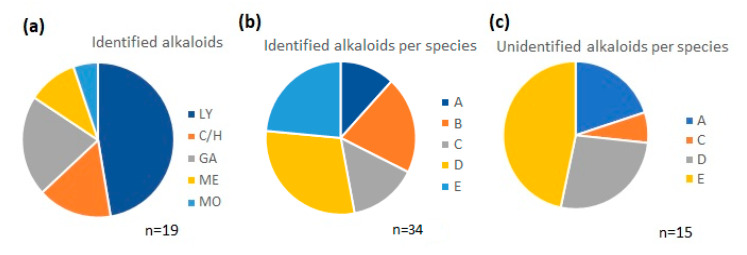
Graphical representation of the chemical profile of *Phaedranassa* Herb. species by GC-MS. (**a**) Distribution of alkaloids identified in all samples by type; (**b**) comparison of number of identified alkaloids among species; (**c**) comparison of number of unidentified alkaloids among species. LY: lycorine-type; C/H: crinine/haemanthamine-type; GA: galanthamine-type; ME: mesembranone-type; MO: montanine-type; A: *P. cinerea*; B: *P. cuencana*; C: *P. dubia*; D: *P. glauciflora*; E: *P. tunguraguae*.

Approximately 50% of the identified alkaloids were of the lycorine type and 15% of the crinine/haemanthamine type. About 85% corresponded to three different alkaloid types—lycorine, crinine/haemanthamine, and galanthamine—and the others were mesembranone- and montanine-type (Figure 3a). The number of alkaloids identified in the samples varied according to the species, ranging from ten in *P. glauciflora* (sample D) to four in *P. cinerea* (sample A) (Figure 3b).

All the alkaloids detected in *P. cuencana* (sample B) were identified (Table 1). The greatest number of unknown compounds (7) was found in *P. tunguraguae* (sample E) (Figure 3c). Overall, we were unable to identify about 40% of the compounds detected in the *Phaedranassa* species, but their structural type was proposed by their fragmentation patterns (Table 1). The high percent of unknown compounds in these samples suggests the genus may contain many new alkaloids. To determine the identity of the unknown structures, it will be necessary to isolate each compound by chromatographic methods and perform a detailed study using spectroscopic methods, such as nuclear magnetic resonance (NMR). In the current study, the quantity of *Phaedranassa* samples was insufficient for this step.

The highest alkaloid concentration was found in *P. cuencana* (sample B) and the lowest in *P. cinerea* (sample A) (191.19 and 51.26 mg GAL·g^−1^ AE, respectively). In about 80% of the samples, lycorine-type alkaloids predominated and were detected in all the species except *P. cinerea* (sample A). Galanthamine-type alkaloids were found in all the species, with the highest concentration in *P. cuencana* (sample B) and the lowest in *P. glauciflora* (sample D) (70.10 and 2.30 mg GAL·g^−1^ AE, respectively). Alkaloids of the crinine/haemanthamine type were detected in 40% of the samples, with the highest concentration in *P. tunguraguae (*7.70 mg GAL·g^−1^ AE). Pancratinine C was the only montanine-type alkaloid identified, present in *P. cinerea* (sample A). Mesembrenone-type alkaloids were detected in low quantities in *P. glauciflora* (5.15 mg GAL·g^−1^ AE) (sample D). The highest content of unidentified alkaloids was detected in *P. tunguraguae* (sample E) (27.54 mg GAL·g^−1^ AE), which had a high concentration of a compound showing an ion peak at *m/z* 275 [M^+^ = 275] (RI 2574.4) (8.87 mg GAL·g^−1^ AE) (Table 1).

A high diversity of lycorine-type alkaloids has also been reported in several *Lycoris* species collected in China, and a great variety of lycorine- and crinine/haemanthamine-type alkaloids have been recently described in different *Rhodophiala* species collected in Chile [16,17]. Previous research has identified phaedranamine, haemanthamine, pseudolycorine, ungeremine, zefbetaine, sanguinine, galanthamine, and epinorgalanthamine alkaloids in a *Phaedranassa dubia* sample collected in Colombia [18]. Moreover, one of the first chemical studies of Amaryllidaceae plants from Ecuador found twenty-two known and five unknown alkaloids in *Stenomesson aurantiacum*, which showed a high abundance of haemanthamine [19].

### 2.2. AChE and BuChE Inhibitory Activities

**Figure 4:**
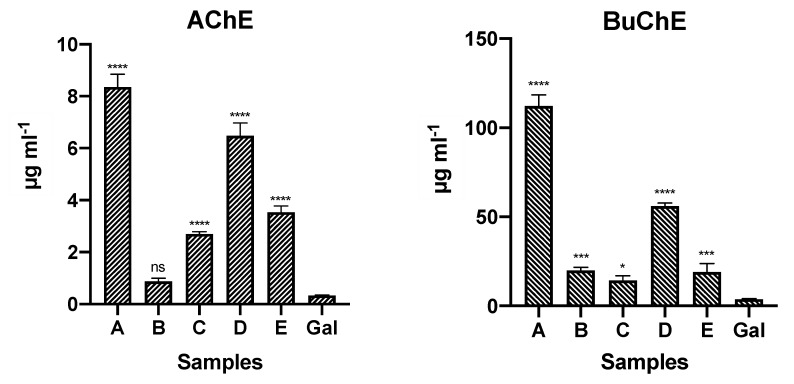
AChE and BuChE inhibitory activities of five different *Phaedranassa* Herb. species from Ecuador. Values are expressed as IC_50_ (µg·mL^−1^). **A**: *P. cinerea*; **B**: *P. cuencana*; **C**: *P. dubia*; **D**: *P. glauciflora*; **E**: *P. tunguraguae*; **GAL**: Galanthamine control. **** *p* < 0.0001, *** *p* < 0.001, * *p* < 0.5, and ns (not significant).

The alkaloid extracts from different species of Ecuadorian *Phaedranassa* Herb. were tested in vitro for AChE and BuChE inhibitory activities (Figure 4). Galanthamine, which was used as a control, presented AChE and BuChE inhibition with IC_50_ values of 0.33 ± 0.02 and 3.81 ± 0.23 µg·mL^−1^, respectively. All the evaluated alkaloid extracts were active against AChE and BuChE, particularly those of *P. cuencana* (sample B), *P. dubia* (sample C), and *P. tunguraguae* (sample E). AChE inhibition was highest in *P. cuencana* (sample B) (IC_50_ value: 0.88 ± 0.11 µg·mL^−1^), whereas the best activity against BuChE was observed in *P. dubia* (sample C) (IC_50_ value: 14.26 ± 2.71 µg·mL^−1^). The high concentration of galanthamine-type alkaloids in *P. cuencana* (sample B) (70.10 mg GAL·g^−1^ AE) could explain its remarkable AChE inhibitory activity.

Galanthamine (**13**) has been described in different Amaryllidaceae genera—including *Haemanthus*, *Lycoris*, *Hippeastrum*, *Hymenocallis*, *Narcissus*, and *Leucojum*—and is obtained from natural sources by pharmaceutical companies [12,20]. Some researchers have reported that the alkaloid sanguinine (**14**) presents higher AChE inhibitory activity than galanthamine (**13**), but they also describe that galanthamine (**13**) can permeate the blood-brain barrier more effectively than sanguinine (**14**) [21]. Studies show that 11α-hydroxy-*O*-methylleucotamine, another galanthamine-type alkaloid, has significant AChE inhibitory activity (IC_50_ 3.5 µM), but no information about its capacity to permeate the blood-brain barrier has been reported [22].

### 2.3. Molecular Docking

The theoretical affinity of all the alkaloids identified in this study toward the active sites of AChE and BuChE together with values reported in the literature are listed in Table 2. The molecular docking results were analyzed for the different interactions between the alkaloids and the active site of the enzymes: hydrogen bonds, π-π stacking, and anionic interactions. For AChE and BuChE, the active site is in the center of the macromolecule, with the catalytic triad region containing residues Ser200, His440, and Glu327 for TcAChE proteins and His438, Ser198, and Glu325 for hBChE proteins [23,24,25].

**Figure 5:**
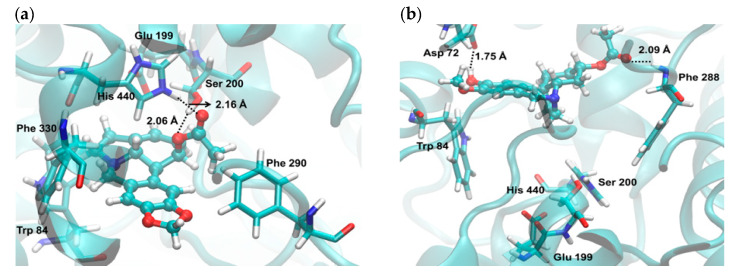
Graphical representations of the binding of (**a**) 1-*O*-acetylcaranine (**2**) and (**b**) cantabricine (**11**) in the gorge of the active site of TcAChE.

No alkaloid identified in the samples presented better theoretical AChE inhibitory activity than galanthamine (**13**). The structures 1-*O*-acetylcaranine (**2**) and cantabricine (**11**) showed high theoretical inhibitory activity against AChE on the 1DX6 structure. The former (**2**) had two strong interactions (hydrogen bonds), with Ser200 at 2.06 and His440 at 2.16 Å (Figure 5a). In a previous in silico study, isoreticulinine—another lycorine-type alkaloid—was identified as a potential cholinesterase inhibitory molecule based on its interaction with the active site of TcAChE and hBuChE through strong hydrogen bonds in both evaluated proteins [26]. It was not possible to verify interactions between cantabricine (**11**) and Ser200, although the cantabricine (**11**)–TcAChE system seems to be stable due to two additional interactions (hydrogen bonds) between this alkaloid and Asp72 at 1.75, and Phe288 at 2.09 Å (Figure 5b).

Molecular simulation of ten alkaloids identified in *Phaedranassa* species on the 4BDS structure theoretically showed higher enzymatic inhibition than galanthamine (**13**) against BuChE. They were 1-*O*-acetylcaranine (**2**), galanthine (**4**), 1-*O*-acetyllycorine (**5**), dihydrolycorine (**6**), lycorine (**7**), sternbergine (**8**), 8-*O*-demethylmaritidine (**10**), cantabricine (**11**), haemanthamine (**12**), and *N*-demethylgalanthamine (**15**). Among them, 1-*O*-acetyllycorine (**5**) and cantabricine (**11**) presented the best estimated binding free energy toward the active site of BuChE, with a theoretical increase of 0.79 and 0.84 kcal·mol^−1^, respectively, compared to galanthamine values. In a recent publication, we also observed that in silico deacetylcantabricine, which has a similar structure to cantabricine (**11**), showed 0.20 kcal·mol^−1^ greater butyrylcholinesterase inhibition on the 4BDS structure compared to galanthamine [17].

**Figure 6:**
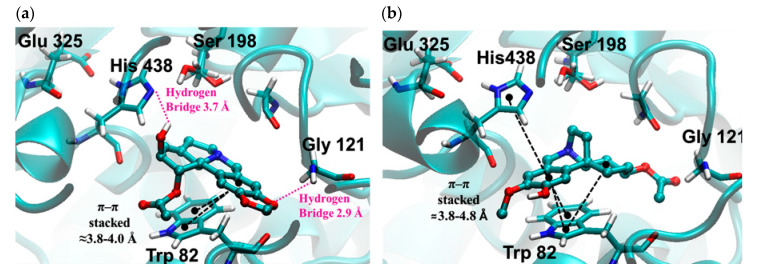
Graphical representations of the binding of (**a**) 1-*O*-acetyllycorine (**5**) and (**b**) cantabricine (**11**) in the gorge of the active site of hBuChE.

The most important molecular interactions of 1-*O*-acetyllycorine (**5**) and cantabricine (**11**) in the gorge of the active site of hBuChE are represented in Figure 6a,b, respectively. The structure of 1-*O*-acetyllycorine (**5**) is stabilized by one π–π stacking interaction with the Trp82 and two hydrogen bridge interactions with Gly121 and His438. In the case of cantabricine (**11**), the stabilization is due to the presence of two π–π stacking interactions with His438 and Trip82. Finally, the molecular docking experiments showed that both alkaloids are close to the catalytic triad of Ser198, Glu325, and His438.

The theoretical experiments indicated that cantabricine (**11**) could be an interesting molecule against both cholinesterases in silico. However, this compound was not detected in *P. cuencana* (sample B) and *P. dubia* (sample C) extracts, which exhibited the best inhibitory activity against AChE and BuChE in vitro. Thus, the high activity of both samples (B and C) could be explained by the presence of galanthamine-type alkaloids, galanthamine (**13**), and *N*-demethylgalanthamine (**15**), with high binding free energy toward the AChE and BuChE cholinesterases (see Table 2). The presence of two alkaloids with a high-energy ligand–protein interaction indicates that the in vitro results could have been enhanced by a synergistic mechanism [25]. The species *P. tunguraguae* (sample E), which does not contain galanthamine-type alkaloids, also presented an AChE and BuChE inhibitory effect in vitro. According to the molecular docking experiments, the presence of cantabricine (**11**) in this sample could have contributed to these results.

## 3. Materials and Methods

### 3.1. Plant Material

Bulbs of five different *Phaedranassa* species were collected in Ecuador in 2017 and 2018. *Phaedranassa cinerea* (Chimborazo, Pallatanga, Ecuador), *Phaedranassa cuencana* (Azuay, Sevilla de Oro, Ecuador), *Phaedranassa dubia* (Pichincha, Pululahua, Ecuador), *Phaedranassa glauciflora* (Chimborazo, Alausí, Ecuador), and *Phaedranassa tunguraguae* (Tungurahua, Baños, Ecuador) (Figure 1). Samples were identified by the group specialist, Dra. Nora Oleas. Herbarium specimen vouchers are *P. cinerea* (Øllgaard and Balslev 9006), *P. cuencana* (Oleas 1031, HUTI), *P. dubia* (Oleas 4, QCA), *P. glauciflora* (Oleas 44, QCA), and *P. tunguraguae* (Oleas 6, QCA).

### 3.2. Extractions

Fresh bulbs of *Phaedranassa* were collected, cleaned, and dried for 7 days at 40 °C. About 2 g of each sample was macerated with MeOH (3 × 100 mL) at room temperature for 3 days. The organic solvent was taken to dryness under reduced pressure to afford the crude extracts, which were acidified to pH 3 with diluted H_2_SO_4_ (2%, *v/v*), and the neutral material was removed with Et_2_O. The aqueous solutions were basified up to pH 10 with NH_4_OH (25%, *v/v*) and extracted with EtOAc to obtain the alkaloid extracts (AE), which were used in the experiments.

### 3.3. Acetylcholinesterase (AChE) and Butyrylcholinesterase (BuChE) Inhibitory Activity

Cholinesterase inhibitory activity was determined according to [27] with some modifications [28]. Stock solutions with 518U of AChE from *Electrophorus electricus* (Merck, Darmstadt, Germany) and BuChE from equine serum (Merck, Darmstadt, Germany), respectively, were prepared and kept at −20 °C. Acetylthiocholine iodide (ATCI), *S*-butyrylthiocholine iodide (BTCI), and 5,5′-dithiobis (2-nitrobenzoic) acid (DTNB) were obtained from Merck (Darmstadt, Germany). Fifty microliters of AChE or BuChE (both enzymes used at 6.24 U) in phosphate buffer (8 mM K_2_HPO_4_, 2.3 mM NaH_2_PO_4_, 0.15 NaCl, pH 7.5) and 50 µL of the sample dissolved in the same buffer were added to the wells. The plates were incubated for 30 min at room temperature. Then, 100 µL of the substrate solution (0.1 M Na_2_HPO_4_, 0.5 M DTNB, and 0.6 mM ATCI or 0.24 mM BTCI in Millipore water, pH 7.5) was added. These reagents were obtained from Merck (Darmstadt, Germany). After 10 min, the absorbance was read at 405 nm in a Labsystem microplate reader (Helsinki, Finland). Enzyme activity was calculated as percent compared to a control using a buffer without any inhibitor. Galanthamine served as a positive control. In a first step, activity of samples was assessed at 10, 100, and 200 µg·mL^−1^ towards both enzymes. Samples with an IC_50_ > 200 µg·mL^−1^ were considered inactive. Samples with an IC_50_ < 200 µg·mL^−1^ were further analyzed to determine the IC_50_ values. The cholinesterase inhibitory data were analyzed with the Microsoft Office Excel 2010 software.

### 3.4. Alkaloid Identification and Quantification

Alkaloid profiles of the *Phaedranassa* samples were obtained using GC-MS (Agilent Technologies 6890N coupled with MSD5975 inert XL; Santa Clara, CA, USA) equipment operating in electron ionization (EI) mode at 70 eV. A Sapiens-X5 MS column (30 m × 0.25 mm i.d., film thickness 0.25 µm; Teknokroma, Barcelona, Spain) was used. The temperature gradient was 12 min at 100 °C, 100–180 °C at 15 °C·min^−1^, 180–300 °C at 5 °C·min^−1^, and 10 min hold at 300 °C. The injector and detector temperatures were 250 and 280 °C, respectively, and the flow-rate of carrier gas (He) was 1 mL·min^−1^. A total of 2 mg of each alkaloid extract was dissolved in 1 mL of MeOH:CHCl_3_ (1:1, *v/v*), and 1 µL of each sample was injected in the equipment using the splitless mode. Codeine (0.05 mg·mL^−1^) was used as the internal standard. Alkaloids were identified by GC-MS and the mass spectra were deconvoluted using the software AMDIS 2.64. Kovats retention indexes (RI) were recorded with a standard calibration n-hydrocarbon mixture (C9-C36) using AMDIS 2.64 software.

A calibration curve of galanthamine (10, 20, 40, 60, 80, and 100 µg·mL^−1^) was applied to quantify each single constituent detected in the chromatogram, using codeine (0.05 mg·mL^−1^) as the internal standard. Peak areas were manually obtained, considering selected ions for each compound (usually the base peak of their MS, i.e., *m/z* at 286 for galanthamine and 299 for codeine). The ratio between the values obtained for galanthamine and codeine in each solution was plotted against the corresponding concentration of galanthamine to obtain the calibration curve and its equation (y = 0.0112x − 0.0469; R^2^ = 0.9995). All data were standardized to the area of the internal standard (codeine), and the equation obtained for the calibration curve of galanthamine was used to calculate the amount of each alkaloid. Results are presented as mg GAL (galanthamine), which was finally related to the alkaloid extract (AE). As the peak area does not only depend on the corresponding alkaloid concentration but also on the intensity of the mass spectra fragmentation, the quantification is not absolute. However, the method is considered suitable to compare the specific alkaloid amount between samples [16,17,29].

### 3.5. Statistical Analysis

Three independent assays were used to evaluate the cholinesterase activity of *Phaedranassa* samples. Results were analyzed by ANOVA, using the PRISM software. Data are expressed as the mean ± standard deviation (SD). Significant results are marked as follows: **** *p* < 0.0001, *** *p* < 0.001, * *p* < 0.5, and *ns* (not significant). One-way ANOVA with Dunnet’s multiple comparison test was used and the differences are with respect to the results of galanthamine with both enzymes.

### 3.6. Molecular Docking

Molecular docking simulations for the principal alkaloids identified from *Phaedranassa* species were performed to investigate the binding mode into the active site of two different enzymes, *Torpedo californica* AChE (TcAChE) and human BuChE (hBuChE), PDB codes 1DX6 [30] and 4BDS [31]. Three-dimensional (3D) structures of the alkaloids were recovered from the PubChem database and submitted to a geometrical optimization procedure at PBE0 [32,33]/6–311 + g* [34] level of theory using the program Gaussian 09 [35]. All optimized alkaloids were confirmed as a minimum on the potential energy surface.

Docking simulations for the set of optimized ligands were performed using the AutoDock v.4.2 program (Molecular Graphics Laboratory, La Jolla, CA, USA) [36]. AutoDock uses a rapid energy evaluation in precalculated grids of affinity potentials and several search algorithms to identify suitable binding positions for a ligand on a given macromolecule. In order to compare the results from the docking simulations, water molecules, cofactors, and ions were excluded from each *x*-ray crystallographic structure. Likewise, polar hydrogen atoms of the enzymes were added and nonpolar hydrogen atoms were merged. Finally, the enzyme was treated as a rigid body. Grid maps of interaction energy for various atom types with each macromolecule were calculated by the auxiliary program AutoGrid (Molecular Graphics Laboratory, La Jolla, CA, USA) using a grid box with dimensions of 60 × 60 × 60 Å around the active site, which was large enough to include the most important residues of each enzyme. Docking searches for the best orientations of the ligands binding to the active site of each protein were performed using the Lamarckian Genetic Algorithm (LGA) [37]. The LGA protocol applied a population size of 2000 individuals, while 2,500,000 energy evaluations were used for the 50 LGA runs. The best conformations were chosen from the lowest docked energy solutions in the cluster populated by the highest number of conformations. Finally, best docking complex solutions (poses) were analyzed according to the potential intermolecular interactions (ligand/enzyme), such as hydrogen bonding and the cation–π, π–π stacking.

## 4. Conclusions

In summary, thirty-three compounds were detected, and nineteen known alkaloids were identified by GC-MS in five different species of *Phaedranassa* Herb. from Ecuador. Galanthamine-type alkaloids were detected in all the samples, with the highest concentration in *P. cuencana*. Extracts from all the species showed activity against AChE and BuChE. In vitro, *P. cuencana* and *P. dubia* proved to be the most active against AChE and BuChE, respectively, whereas in silico results indicated that cantabricine is highly inhibitory against both cholinesterases. This study, which is the first to report the alkaloid profile and biological activities of *P. cuencana*, *P. glauciflora*, and *P. tunguraguae*, supports the role of Amaryllidaceae species as a source of alkaloids with potential application for the palliative treatment of AD.

## Figures and Tables

**Table 1:** Alkaloids identified in different species of *Phaedranassa* Herb. from Ecuador by GC-MS. Values are expressed as mg GAL·g^−1^ AE.

Alkaloid	[M^+^]	B.P.	R.I.	A *^a^	B *^b^	C *^c^	D *^d^	E *^e^
Lycorine-type				-	121.09	85.21	114.18	85.34
Anhydrolycorine (**1**)	251	250	2534.4	-	2.39	3.52	2.69	-
1-*O*-Acetylcaranine (**2**)	313	252	2554.2	-	-	-	2.32	-
11,12-Dehydroanhydrolycorine (**3**)	249	248	2638.6	-	2.39	3.43	2.80	-
Galanthine (**4**)	317	242	2730.1	-	-	-	-	5.36
1-*O*-Acetyllycorine (**5**)	329	226	2747.2	-	2.59	-	2.35	-
Dihydrolycorine (**6**)	289	288	2781.5	-	-	-	-	11.80
Lycorine (**7**)	287	226	2795.8	-	113.72	78.26	93.92	57.04
Sternbergine (**8**)	331	228	2831.4	-	-	-	10.10	8.64
2-Methoxypratosine (**9**)	309	309	3084.0	-	-	-	-	2.50
Crinine/haemanthamine-type				-	-	-	6.34	7.70
8-*O*-Demethylmaritidine (**10**)	273	273	2532.2	-	-	-	-	3.81
Cantabricine (**11**)	317	317	2639.9	-	-	-	-	3.89
Haemanthamine (**12**)	301	272	2668.8	-	-	-	6.34	-
Galanthamine-type				27.87	70.10	15.80	2.30	2.33
Galanthamine (**13**)	287	286	2385.2	15.99	48.60	7.16	2.30	2.33
Sanguinine (**14**)	273	273	2403.1	-	18.85	-	-	-
*N*-Demethylgalanthamine (**15**)	273	272	2428.3	5.51	2.65	8.64	-	-
*N*-Formylnorgalanthamine (**16**)	301	301	2834.4	6.37	-	-	-	-
Mesembranone-type				-	-	-	5.15	-
6-Epimesembranol (**17**)	291	290	2379.7	-	-	-	2.90	-
Mesembrine (**18**)	289	218	2396.5	-	-	-	2.25	-
Montanine-type				6.17	-	-	-	-
Pancratinine C (**19**)	287	176	2622.2	6.17	-	-	-	-
Unidentified				17.22	-	2.40	11.76	27.54
Unknown (**20**) (homolycorine type) *^g^	301 *^f^	109	2500.8	-	-	-	-	2.62
Unknown (**21**)	371 *^f^	130	2513.4	5.76	-	-	-	-
Unknown (**22**) (lycorine type) *^g^	315 *^f^	228	2544.5	-	-	-	3.23	-
Unknown (**23**)	239 *^f^	239	2548.8	5.86	-	-	-	-
Unknown (**24**) (crinine/haem. type) *^g^	275 *^f^	275	2574.4	-	-	-	-	8.87
Unknown (**25**) (lycorine type) *^g^	265 *^f^	264	2707.4	5.60	-	-	-	3.00
Unknown (**26**) (lycorine type) *^g^	331 *^f^	228	2738.0	-	-	-	3.37	-
Unknown (**27**) (lycorine type) *^g^	303 *^f^	228	2771.7	-	-	-	-	3.32
Unknown (**28**) (lycorine type) *^g^	315 *^f^	240	2805.7	-	-	-	-	2.26
Unknown (**29**) (lycorine type) *^g^	329 *^f^	268	2873.2	-	-	2.40	-	-
Unknown (**30**) (lycorine type) *^g^	289 *^f^	228	2873.9	-	-	-	2.81	-
Unknown (**31**) (homolycorine type) *^g^	331 *^f^	125	2928.3	-	-	-	2.35	-
Unknown (**32**) (lycorine type) *^g^	374 *^f^	284	2953.7	-	-	-	-	3.04
Unknown (**33**) (lycorine type) *^g^	295 *^f^	294	2963.1	-	-	-	-	4.43
Total				51.26	191.19	103.41	139.73	122.91

*^a^ **A**: *P. cinerea*; *^b^ **B**: *P. cuencana*; *^c^ **C**: *P. dubia*; *^d^ **D**: *P. glauciflora*; *^e^ **E**: *P. tunguraguae*; *^f^ possible molecular ion peak; *^g^ proposed structure-type according to the fragmentation pattern.

**Table 2:** Estimated binding free energy in molecular docking of alkaloids identified in extracts of *Phaedranassa* Herb. species toward cholinesterases (AChE and BuChE). Values expressed in kcal·mol^−1^.

Alkaloid	AChE	BuChE	Reference
Lycorine-Type			
Anhydrolycorine (**1**)	−8.38 ^a^; −8.35 ^c^	−8.14 ^b^	[25]
1-*O*-Acetylcaranine (**2**)	−9.55 ^a^	−8.78 ^b^	Calculated values
11,12-Dehydroanhydrolycorine (**3**)	−8.41 ^a^	−7.44 ^b^	[17]
Galanthine (**4**)	−8.43 ^a^	−8.37 ^b^	Calculated values
1-*O*-Acetyllycorine (**5**)	−8.82 ^a^	−9.02 ^b^	Calculated values
Dihydrolycorine (**6**)	−8.76 ^a^; −9.07 ^c^	−8.80 ^b^	[25]
Lycorine (**7**)	−8.82 ^a^	−8.94 ^b^	[17]
Sternbergine (**8**)	−8.61 ^a^	−8.71 ^b^	Calculated values
2-Methoxypratosine (**9**)	−8.14 ^a^	−7.81 ^b^	Calculated values
**Homolycorine-type**			
**Crinine/haemanthamine-type**			
8-*O*-Demethylmaritidine (**10**)	−8.74 ^a^	−8.93 ^b^	[17]
Cantabricine (**11**)	−9.15 ^a^	−9.07 ^b^	Calculated values
Haemanthamine (**12**)	−8.80 ^a^	−8.34 ^b^	[17]
**Galanthamine-type**			
Galanthamine (**13**)	−10.10 ^a^	−8.23 ^b^	Calculated values
Sanguinine (**14**)	−9.40 ^a^	−7.92 ^b^	[25]
*N*-Demethylgalanthamine (**15**)	−9.09 ^a^	−8.81 ^b^	Calculated values
*N*-Formylnorgalanthamine (**16**)	−8.72 ^a^	−8.12 ^b^	Calculated values
**Mesembranone-type**			
6-Epimesembranol (**17**)	−8.15 ^a^	−7.59 ^b^	Calculated values
Mesembrine (**18**)	−8.77 ^a^	−8.14 ^b^	Calculated values
**Montanine-type**			
Pancratinine C (**19**)	−8.53 ^a^	−8.12 ^b^	Calculated values

^a^ PDB code: 1DX6; ^b^ PDB code: 4BDS; ^c^ PDB code: 4EY7.

## References

[1] Newman D.J., Cragg G.M. (2016). Natural products as sources of new drugs from 1981 to 2014. J. Nat. Prod..

[2] Harvey A. (2000). Strategies for discovering drugs from previously unexplored natural products. Drug Discov. Today.

[3] Rodrigues T., Reker D., Schneider P., Schneider G. (2016). Counting on natural products for drug design. Nat. Chem..

[4] Stratton C.F., Newman D.J., Tan D.S. (2015). Cheminformatic comparison of approved drugs from natural products versus synthetic origins. Bioorg. Med. Chem. Lett..

[5] Bastida J., Lavilla R., Viladomat F., Cordell G.A. (2006). Chemical and biological aspects of *Narcissus* Alkaloids. The Alkaloids: Chemistry and Physiology.

[6] Minga D., Ulloa C.U., Oleas N., Verdugo A. (2015). A new species of *Phaedranassa* (Amaryllidaceae) from Ecuador. Phytotaxa.

[7] Oleas N. (2011). Landscape Genetics of *Phaedranassa* Herb. (Amaryllidaceae) in Ecuador. Ph.D. Thesis.

[8] Oleas N., León-Yánez S., Valencia R., Pitman N., Endara L., Ulloa Ulloa C., Navarrete H. (2011). Amaryllidaceae. Libro Rojo de Plantas Endémicas del Ecuador.

[9] Oleas N.H., Meerow A.W., Francisco-Ortega J. (2012). Population dynamics of the endangered plant, *Phaedranassa tunguraguae*, from the Tropical Andean Hotspot. J. Hered.

[10] Oleas N.H., Meerow A.W., Francisco-Ortega J. (2016). Genetic structure of the threatened *Phaedranassa schizantha* (Amaryllidaceae). Bot. J. Linn. Soc..

[11] (2018). Alzheimer’s Disease International. https://www.alz.co.uk/research/WorldAlzheimerReport2018.pdf.

[12] Selkoe D.J. (2001). Alzheimer’s Disease: Genes, proteins, and therapy. Physiol. Rev..

[13] Konrath E.L., dos Santos Passos C., Klein-Júnior L.C., Henriques A.T. (2013). Alkaloids as a source of potential anticholinesterase inhibitors for the treatment of Alzheimer’s disease. J. Pharm. Pharm..

[14] Maelicke A., Samachocki M., Jostock R., Fehrenbacher A., Ludwig J., Albuquerque E.X., Zerlin M. (2001). Allosteric sensitization of nicotine receptors by galanthamine, a new treatment strategy for Alzheimer’s disease. Biol. Psychiatry.

[15] Torras-Claveria L., Tallini L., Viladomat F., Bastida J., Muñoz-Torrero D., Riu M., Feliu C. (2017). Research in natural products: Amaryllidaceae ornamental plants as sources of bioactive compounds. Recent Advances in Pharmaceutical Sciences VII.

[16] Guo Y., Pigni N.B., Zheng Y., de Andrade J.P., Torras-Claveria L., Borges W.S., Viladomat F., Codina C., Bastida J. (2014). Analysis of bioactive Amaryllidaceae alkaloid profiles in *Lycoris* species by GC-MS. Nat. Prod. Commun..

[17] Tallini L.R., Bastida J., Cortes N., Osorio E.H., Theoduloz C., Schemeda-Hirschmann G. (2018). Cholinesterase inhibition activity, alkaloid profiling and molecular docking of Chilean *Rhodophiala* (Amaryllidaceae). Molecules.

[18] Osorio E.J., Berkov S., Brun R., Codina C., Viladomat F., Cabezas F., Bastida J. (2010). In vitro antiprotozoal activity of alkaloids from *Phaedranassa dubia* (Amaryllidaceae). Phytochem. Lett..

[19] Acosta K., Pigni N., Oleas N., Bastida J. (2014). Identification of the alkaloids of *Stenomesson aurantiacum* (Kunth) Herb., an Amaryllidaceae species from the Ecuadorian Andes. Pharmacol. Online.

[20] Berkov S., Georgieva L., Kondakova V., Atanassov A., Viladomat F., Bastida J., Codina C. (2009). Plant sources of galanthamine: Phytochemical and biotechnological aspects. Biotechnol. Biotechnol. Equip..

[21] Bores G.M., Huger F.P., Petko W., Mutlib A.E., Camacho F., Rush D.K., Selk D.E., Wolf V., Kosley R.W., Davis L. (1996). Pharmaceutical evaluation of novel Alzheimer’s disease therapeutics: Acetylcholinesterase inhibitors related to galanthamine. J. Pharm. Exp..

[22] Iannello C., Pigni N.B., Antognoni F., Poli F., Maxia A., de Andrade J.P., Bastida J. (2014). A potent acetylcholinesterase inhibitor from *Pancratium illyricum* L. Fitoterapia.

[23] Sussman J.L., Harel M., Frolow F., Oefner C., Goldman A., Toker L., Silman I. (1991). Atomic structure of acetylcholinesterase from *Torpedo californica*: A prototypic acetylcholine-binding protein. Science.

[24] Nicolet Y. (2003). Crystal structure of human butyrylcholinesterase and of its complexes with substrate and products. J. Biol. Chem..

[25] Cortes N., Sierra K., Alzate F., Osorio E.H., Osorio E. (2018). Alkaloids of Amaryllidaceae as inhibitors of cholinesterases (AChEs and BuChEs): An integrated bioguided study. Phytochem. Anal..

[26] Tallini L.R., Osorio E.H., dos Santos V.D., De Souza Borges W., Kaiser M., Viladomat F., Zuanazzi J.A.S., Bastida J. (2017). *Hippeastrum reticulatum* (Amaryllidaceae): Alkaloid, profiling, biological activities and molecular docking. Molecules.

[27] Ellman G.L., Courtney K.D., Andres Jr. V., Featherstone R.M. (1961). A new and rapid colorimetric determination of acetylcholinesterase activity. Biochem. Pharm..

[28] López S., Bastida J., Viladomat F., Codina C. (2002). Acetylcholinesterase inhibitory activity of some Amaryllidaceae alkaloids and *Narcissus* extracts. Life Sci..

[29] Torras-Claveria L., Berkov S., Codina C., Viladomat F., Bastida J. (2014). Metabolomic analysis of bioactive Amaryllidaceae alkaloids of ornamental varieties of *Narcissus* by GC-MS combined with k-means cluster analysis. Ind. Crop. Prod..

[30] Greenblatt H.M., Kryger G., Lewis T., Silman I., Sussman J.L. (1999). Structure of acetylcholinesterase complexed with (-)-galanthamine at 2.3 Å resolution. Febs Lett..

[31] Nachon F., Carletti E., Ronco C., Trovaslet M., Nicolet Y., Jean L., Renard P.-Y. (2013). Crystal structures of human cholinesterases in complex with huprine W and tacrine: Elements of specificity for anti-Alzheimer’s drugs targeting acetyl- and butyryl-cholinesterase. Biochem. J..

[32] Adamo C., Barone V. (1999). Toward reliable density functional methods without adjustable parameters: The PBE0 model. J. Chem. Phys..

[33] Ernzerhof M., Scuseria G.E. (1999). Assessment of the Perdew–Burke– Ernzerhof exchange-correlation functional. J. Chem. Phys..

[34] Petersson G.A., Bennett A., Tensfeldt T.G., Al-Laham M.A., Shirley W.A., Mantzaris J., Mantzaris J. (1988). A complete basis set model chemistry. I. The total energies of closed-shell atoms and hydrides of the first-row elements. J. Chem. Phys..

[35] Frisch M.J., Trucks G.W., Schlegel H.B., Scuseria G.E., Robb M.A., Cheeseman J.R., Scalmani G., Barone V., Mennucci B., Petersson G.A. (2013). Gaussian 09, Revis. E.01.

[36] Moris G.M., Huey R., Lindstrom W., Sanner M.F., Belew R.K., Goodsell D.S., Olson A.J. (2009). Autodock4 and AutoDockTools4: Automated docking with selective receptor flexibility. J. Comput. Chem..

[37] Thomsen R., Christensen M.H. (2006). MolDock: A new technique for high-accuracy molecular docking. J. Med. Chem..

